# Community antibiotic consumption and associated factors in Lusaka district of Zambia: findings and implications for antimicrobial resistance and stewardship

**DOI:** 10.1093/jacamr/dlae034

**Published:** 2024-03-05

**Authors:** Maty Tsumbu Ngoma, Doreen Sitali, Steward Mudenda, Mercy Mukuma, Flavien Nsoni Bumbangi, Emmanuel Bunuma, Eystein Skjerve, John Bwalya Muma

**Affiliations:** Department of Disease Control, School of Veterinary Medicine, University of Zambia, Lusaka, Zambia; Department of Health Promotion, School of Public Health, University of Zambia, Lusaka, Zambia; Department of Pharmacy, School of Health Sciences, University of Zambia, Lusaka, Zambia; Department of Food Science, School of Agricultural Sciences and Nutrition, University of Zambia, Lusaka, Zambia; Department of Medicine and Clinical Sciences, School of Medicine, Eden University, Lusaka, Zambia; Department of Biomedical Sciences, School of Veterinary Medicine, University of Zambia, Lusaka, Zambia; Faculty of Veterinary Medicine, Norwegian University of Life Sciences, Ås, Norway; Department of Disease Control, School of Veterinary Medicine, University of Zambia, Lusaka, Zambia

## Abstract

**Introduction:**

Antimicrobial resistance (AMR) is a global public health crisis. This study assessed the general public’s consumption of antibiotics and associated factors in the Lusaka district of Zambia.

**Methods:**

This cross-sectional study was conducted among 2038 participants between December 2022 and January 2023. Data were analysed using Stata 13.0. Multivariable regression techniques were used to determine the factors that influenced antibiotic consumption.

**Results:**

Of the 2038 participants, 53.4% were female, and 51.5% had attended at least secondary school. Antibiotic use was 99.2%, of which 40.9% were appropriately used. Overall, 79.1% of antibiotics were prescribed in hospitals, while 20.9% were used from leftovers and accessed without prescriptions. This study found that the appropriate use of antibiotics was associated with being female, being aged 35 years and above, attaining secondary school or tertiary education, having a monthly expenditure of 195 USD and above, being aware that antibiotics were not the same as painkillers, and being confident that when someone was hospitalized, they would get well.

**Conclusions:**

This study found that the appropriate use of antibiotics was low, and this is an urgent public health issue requiring community engagement in tackling AMR and adherence to treatment guidelines in healthcare facilities. Additionally, there is a need to implement and strengthen antimicrobial stewardship programmes in healthcare facilities to promote the rational use of antibiotics in Zambia. There is also a need to heighten community awareness campaigns and educational activities on the appropriate use of antibiotics.

## Introduction

Antibiotics are medicines used to prevent and treat bacterial infections in humans, animals and plants.^1,2^ Unfortunately, their inappropriate use is among the major contributing factors to developing antimicrobial resistance (AMR).^3–8^ AMR is a common public health issue worldwide and is currently referred to as a silent pandemic.^5,9–14^ It poses a serious threat to all advances in modern medicine, the success of which depends on antimicrobials, causing increased mortality, longer hospital stays and higher medical expenses.^5,10,15–17^ Consequently, if AMR is not addressed, it is projected that approximately 10 million people more will die annually and an estimated 24 million more will live in extreme poverty by the year 2050.^18–20^

AMR is an inevitable natural phenomenon but accelerated by the high consumption and misuse of antimicrobials.^21,22^ This may happen through antimicrobial overprescribing or underprescribing by prescribers, empirical treatment, self-medication and purchasing drugs without a legal prescription.^10,23–27^ Some determinants affect the choice and attitude towards antimicrobial use (AMU).^28–31^ Some factors influence individuals’ choices and attitudes toward AMU, including readily accessible retail pharmacies, which have emerged as the predominant source of outpatient care in Africa.^28,32^ Other determinants of AMU include gender, as well as the individual’s level of knowledge and attitude.^33^ Other factors contributing to AMR’s emergence include a lack of diagnostic tools in hospitals, a lack of patient education on AMU and AMR, inadequate antibiotic regulatory mechanisms, and the unauthorized sale of antimicrobials.^21^ Further, the overuse and misuse of antimicrobials in the animal sector also contribute to the emergence and spread of antimicrobial-resistant infections.^34–36^ This has been demonstrated by an increased number of pathogens that have developed resistance to antimicrobials, some of which are of human priority.^37–41^

The selling of antimicrobials, especially antibiotics, is recognized as one of the more profitable businesses, especially in the private health sector.^42–44^ This sector is well developed in low-and middle-income countries (LMICs),^45^ but it is not well regulated and is frequently neglected by governments, even though private pharmacies are perceived as the community’s first source of healthcare.^21,46^ This situation constitutes the main reason for the high consumption of antibiotics and, consequently, may contribute to the extensive selection of resistant bacteria in the community.^47–49^ This is because there is a link between antibiotic consumption and AMR.^50–53^ Despite this understanding, the misuse and overuse of antibiotics persist globally in both healthcare facilities and the community because of the complex interplay between profitability, regulation and the public health challenges associated with antibiotic use.^23,54,55^

The inappropriate prescription of antimicrobials is tied to the prescribers’ lack of knowledge and attitudes towards the rational use of antimicrobials, and physicians can be forced to prescribe antibiotics for conditions like upper respiratory tract infections (URTIs) due to the perceived effectiveness of these drugs, even when the evidence indicates that antibiotics may not be necessary.^56–60^ This pressure is noted to be significant in the context of South Africa, among other African countries.^23,56,61,62^ Additionally, the inappropriate use of antibiotics has been reported to be due to a lack of: diagnostic tests; poor infection, prevention and control (IPC) practices; insufficient funding; poor vaccine coverage and uptake; illegal drug stores; substandard antibiotics; irresponsible use of laboratory services by clinicians; and a lack of political will.^31,63–65^ The most inappropriate use of antibiotics is observed in cases of: URTIs; generally self-limited viral infections; acute diarrhoea; and urinary tract infections.^21,66,67^ The inappropriate prescription of antimicrobials leads to increased AMR and mortality, morbidity and healthcare costs.^68^ Moreover, inappropriate antibiotic use has economic repercussions, particularly concerning URTIs in the African context.^66,69^ The escalated costs imply that the inappropriate use of antibiotics not only adds to health-related challenges but also carries economic implications.^69^

The lack of surveillance on antimicrobial use implies that the breadth of AMR issues in Africa needs to be better documented.^70^ Currently, there are inadequate updated data on AMR in African countries, with only 42.6% of the countries reported to have documented some data.^71^ Ample data on the burden of infections caused by antibiotic-resistant bacteria in developed countries, such as the EU, reveal approximately 33 110 (28 480–38 430) deaths and 874 541 (768 837–989 068) disability-adjusted life years (DALYs) are attributed to the resistant microbes,^72^ whilst the insufficient data on AMR in Zambia reveal that a significant number of pathogens resistant to the most commonly prescribed antibiotics may be circulating in the communities.^71,73^

Addressing the factors that lead to AMR requires developing and implementing antimicrobial stewardship (AMS) programmes.^74–83^ AMS programmes are effective in promoting the rational use of antibiotics among community members.^77–79,84^ Therefore, community engagement through antibiotic awareness campaigns is critical in addressing some factors contributing to the overuse and misuse of antibiotics in communities.^85–88^ Additionally, community-based educational programmes must also focus on behavioural change in the use of antibiotics.^74,89–92^ Furthermore, the gravity of this issue is underscored by a recent report from the Organisation for Economic Co-operation and Development (OECD).^93^ The report predicts that over the next 30 years, 2.4 million individuals in Europe, North America and Australia could die from infections caused by resistant microorganisms, incurring an annual cost of up to 3.5 billion USD.^93^ Additionally, many LMICs, including Zambia, already grapple with elevated resistance rates, projected to escalate disproportionately. Consequently, the anticipated cost of AMR in these nations might surpass that projected for the developed countries mentioned earlier, given that there is still a high percentage of surveyed pharmacists who still dispense antibiotics without a prescription.^94,95^

Zambia is a country in sub-Saharan Africa with a high burden of infectious diseases.^73,96–103^ Additionally, there is evidence of misuse and overuse of antibiotics among Zambian communities.^94,104–108^ Consequently, there is evidence of antimicrobial-resistant pathogens across the human and animal sectors.^37,38,41,99,109–115^ A study by Masich *et al.*^116^ revealed that about 67% of antimicrobials were inappropriately prescribed to non-critically ill adult patients admitted to the University Teaching Hospital in Lusaka, Zambia. Intriguingly, the Zambian government has put in measures to promote the appropriate use of antibiotics by developing and implementing the National Action Plan (NAP) on AMR.^117,118^ The Antimicrobial Resistance Coordinating Committee (AMRCC) has been coordinating activities to educate the general public on the appropriate use of antibiotics and AMR. However, there is a paucity of information concerning the appropriate consumption of antibiotics among the general public in Zambian communities. Therefore, this study assessed antibiotic consumption and associated factors among residents in selected communities of Lusaka district, Zambia.

## Materials and methods

### Study design, setting and population

This cross-sectional study was conducted between December 2022 and January 2023 among residents of Lusaka district in Zambia. A cross-sectional study design was chosen because this was a population-based survey and it allowed for collection of data from the participants during the same period to avoid discrepancies in the findings. Lusaka district was selected because it is the most developed and well-represented population of different ethnicities and communities of different socioeconomic backgrounds and constitutes an excellent attraction factor for any commercial activity. Furthermore, many pharmacies in Lusaka provide healthcare services to the communities.^105^ The national population of Zambia is agglomerated essentially around Lusaka Province in the south and Copperbelt Province in the North, the two core economic hubs of the country.^119^ With a total area of 21 896 km^2^, Lusaka Province is Zambia’s smallest province but the most densely populated and urbanized province. Lusaka Province has a population of about 3 million people and a density of 140.1 people/km^2^ (Census of Zambia, 2022).^120^ The province is divided into six districts: Lusaka (population: 2 204 059), Chilanga (population: 225 276), Chongwe (population: 313 389), Kafue (population: 219 574), Luangwa (population: 35 933) and Rufunsa (population: 81 733).^120^ Based on existing geopolitical structure, population size and covered health services, Lusaka district was divided into 11 study areas: Mtendere, Kaunda Square, Chelstone, Kalingalinga, Chainda, Chipata, Ng’ombe, Matero, George, Kanyama and Chawama. Lusaka Province is located in the south-central part of the country.

### Study population and sample size estimation

The sampling sites represented 11 communities with approximately 10 000 people in each area. We used Cochrane’s formula to estimate the sample size.^121^ Given a 33% expected appropriate use of antibiotics, as reported earlier,^116^ and a margin of error of 5%, we estimated a minimum sample size of 340 participants. All the participants were selected using stratified and simple random sampling methods. Each area contained at least one health centre and health posts serving approximately 10 000 residents. To be eligible, a participant was an adult resident of the selected communities in the Lusaka district and provided consent to participate in the study. Therefore, this study excluded all respondents under 18 years old and those who had not resided in Lusaka for at least a year. Individuals who did not use antibiotics in the last 12 months during the data collection period were also excluded from the study. All participants were first grouped into their respective community areas. This was followed by sampling each participant from randomly selected households.

### Data collection

Data collection was done using a structured questionnaire (Supplementary data S1, available as Supplementary data at *JAC-AMR* Online). The questionnaire was reviewed for content and face validity by experts from the University of Zambia. Before the main study, a pilot study was undertaken in October 2022, and a sample of 482 participants was collected only from shopping malls, churches, streets, markets, parking areas and healthcare facilities within the selected study areas, which allowed the researchers to understand important variations among the population of the Lusaka district and validated the data collection tool. The findings of the pilot study were excluded from the main study findings. In the main study, from the randomly selected households, a simple random sampling method was used to select participants who were interviewed face to face using a structured questionnaire with Epicollect5 software (https://five.epicollect.net/). The questionnaire was designed in English with three sections: site; sociodemographic information of the participants; access to antibiotics and pattern of antibiotic use. The maximum time for the interview was approximately 20 to 30 min.

### Data management and analysis

The data collected from Epicollect5 were imported into a Microsoft Excel spreadsheet version 2013 for data cleaning. The cleaned data were transferred to STATA version 17.0 for descriptive and statistical analysis. The outcome variable was appropriate antibiotic use by the study participants. In this study, appropriate antibiotic use was defined as obtaining antibiotics through a prescription written by a qualified prescriber and completing the course of antibiotic therapy as recommended. Univariable analysis was used to determine the relationship between residents’ antibiotic consumption and explanatory variables, gender, age, level of education, marital status, monthly income, being aware that antibiotics were not the same as painkillers, and confidence that an admitted patient would get well. In the first step, analysis was performed to identify important covariates; we fitted one predictor variable at a time, using the chi-squared test or, where necessary, the Fisher’s exact test, to establish potential determinants of appropriate antibiotic consumption. After that, the candidate variables were selected based on the *P* value cut-off point of 0.25, which is a purposeful selection of the algorithm as proposed by Hosmer and Lemeshow,^122^ whereas those risk factors with *P* > 0.25 were left out as having no significant effect on the outcome. The logistic regression model was built with variables selected in step 1 through a backward selection strategy, using a *P* value of <0.05 of the likelihood ratio test as inclusion criteria.

## Ethics

Ethical approval for this study was sought from ERES CONVERGE IRB, approval Ref. No. 2022-Mar-020. Regulatory approval was obtained from the National Health Research Authority (NHRA) with an approval number of NHRA0000016/31/102022. Participation in the study was voluntary after providing informed and written consent.

## Results

### Demographic information of the participants

A total of 2038 Lusaka residents were enrolled in this study, of which 53.4% were female and the majority (27.6%) aged between 31 and 35 years. The number of respondents interviewed from each area varied between 146 and 221. Most of the population (57.8%) were married, 72.1% had attended at least secondary school, and 91.9% spent below 195 USD per month (Table 1).

**Table 1.** Sociodemographic characteristics of study participants

Variable	Variable level/group	Frequency	(%)
Residential area	Mtendere	159	7.80
	Kaunda square	208	10.20
	Chelstone	166	8.10
	Kalingalinga	177	8.70
	Chainda	221	10.80
	Chipata	187	9.20
	Ng’ombe	179	8.80
	Matero	201	9.90
	George	195	9.60
	Kanyama	199	9.80
	Chawama	146	7.10
Gender	Male	950	46.60
	Female	1088	53.40
Age group (years)	18–25	476	23.40
	26–30	461	22.60
	31–35	563	27.60
	Above 35	538	26.40
Marital status	Unmarried	860	42.20
	Married	1178	57.80
Level of education	Up to primary level	568	27.90
	Secondary and above	1470	72.10
Monthly expenditure	Below 195 USD	1874	91.90
	195 USD and above	164	8.10

### Antibiotic acquisition information

Most residents (73.2%) obtained their antibiotics from healthcare facilities (hospitals and clinics), and 79.1% of these participants purchased antibiotics at the pharmacy using a medical prescription (Table 2). Few (17/2038) residents claimed never to have used antibiotics in the last 12 months.

**Table 2.** Mode of access to antibiotics among study participants

Variable	Group	Frequency (*n*) (*N* = 2038)	(%)
Where did you obtain your last antibiotic?	NA (neither suffered nor use of antibiotic)	17	0.8
	Health facility	1495	73.2
	Leftover antibiotics from the previous treatment	169	8.3
	Pharmacy	338	16.5
	Do not know or do not remember	24	1.2
How did you obtain your last antibiotic used?	Prescribed by a doctor or a clinical officer and dispensed by a pharmacist	1598	79.1
	Recommended and supplied by a pharmacist or drug retailer without a prescription	310	15.3
	Self-medicated (you indicate to the pharmacist what drug you want)	113	5.6

### Pattern of antibiotic consumption among residents of Lusaka district

Out of the 2021 participants who used antibiotics, 921 (45.6%) used antibiotics correctly, although 95 (4.7%) did not obtain their antibiotics properly. Thus, the proportion of appropriate use of antibiotics is estimated at 40.9% (those who obtained antibiotics using prescriptions and used them as guided), while 59.1% accounted for inappropriate use of antibiotics (Table 3).

**Table 3.** Antibiotic consumption patterns among study participants

When did you stop taking the last antibiotics you purchased? (*N* = 2021)	How did you obtain the last antibiotics you took?		Total
	Prescribed by a doctor/clinical officer and dispensed by a pharmacy professional, *n* (%)	Not prescribed by a doctor or a clinical officer, *n* (%)	
When my illness was better	741 (36.7)	323 (16.0)	1064
When I got a full course as prescribed by a doctor or clinical officer	826 (40.9)	95 (4.7)	921
I do not remember	31 (1.5)	5 (0.3)	36
Total	1598	423	2021

### Antibiotic consumption among residents of Lusaka district with associated factors

The univariable analysis (cross-tabulation between predictor and outcome variable) of the sociodemographic characteristics versus antibiotic consumption among residents of the Lusaka district showed that all the independent variables examined were significantly associated with the study outcome (Table 4).

**Table 4.** Factors associated with appropriate antibiotic consumption among community members of Lusaka district on univariable analysis

Characteristic	Total	Taken as prescribed and recommended by the prescriber		Not prescribed nor taken as recommended by the prescriber		*P* value
	N	*n*	%	*n*	%	
Total	2038	829	40.7	1209	59.3	
Gender						<0.001
Male	950	343	16.8	607	29.8	
Female	1088	486	23.9	602	29.5	
Age group (years)						<0.001
18–25	476	163	8.0	313	15.4	
26–30	461	158	7.8	303	14.9	
31–35	563	219	10.8	344	16.9	
Above 35	538	289	14.2	249	12.2	
Marital status						<0.037
Unmarried	860	327	16.1	533	26.1	
Married	1178	502	24.6	676	33.2	
Level of education						<0.001
Up to primary level	568	108	5.3	460	22.6	
Secondary and above	1470	721	35.4	749	36.7	
Monthly expenditure						<0.001
Below 195 USD	1874	736	31.1	1138	60.8	
195 USD and above	164	93	4.6	71	3.5	
Antibiotics are the same as Dolaren, Paracetamol, Diclofenac						0.001
True	182	59	2.9	123	6.0	
False	1366	540	26.5	826	40.5	
I do not know	490	230	11.3	260	12.8	
When a patient is admitted to a hospital						<0.001
I am confident they will get well	694	256	12.6	438	21.5	
Not confident	1173	526	25.8	647	31.7	
It depends on the sickness	124	22	1.1	102	5.0	
I do not know	47	25	1.2	22	1.1	

### Factors affecting appropriate consumption of antibiotics among study participants

The multivariable logistic regression model results showed that the appropriate use of antibiotics was related to gender. The appropriate use of antibiotics was associated with being female, age above 35 years, attaining secondary school or tertiary education, having a monthly expenditure of 195 USD and above, being aware that antibiotics were not the same as painkillers like paracetamol and diclofenac, and being aware of the disease that a patient suffered made Lusaka residents confident that someone will get well after being admitted to hospital (Table 5). Females were more likely (OR = 1.4) to use antibiotics appropriately than males. Additionally, participants who were aged above 35 years of age were more likely (OR = 2.1) to use antibiotics compared with those who were aged between 18 and 25 years. Further, participants who attained secondary school or tertiary education were more likely (OR = 4.6) to use antibiotics appropriately compared with those who had only reached primary school level. Furthermore, participants who had a monthly expenditure of 195 USD and above were more likely to use antibiotics appropriately than those who spent less than 195 USD per month. Our study also revealed that those who were aware that antibiotics were not the same as painkillers were more likely (OR = 1.5) to use antibiotics appropriately than those who thought antibiotics were the same as painkillers.

**Table 5.** Factors associated with appropriate antibiotic consumption among community members of Lusaka district based upon a multivariable logistic model

Variable	Characteristics	Adjusted OR	95% CI	*P* value
Gender	Male	1		
	Female	1.4	1.17–1.73	<0.001
Age (years)	18–25	1		
	26–30	0.8	0.61–1.08	0.155
	31–35	1.1	0.84–1.46	0.454
	Above 35	2.1	1.58–2.74	<0.001
Level of education	Up to primary level	1		
	Secondary and above	4.6	3.53–5.86	<0.001
Monthly expenditure	Below 195 USD	1		
	195 USD and above	1.9	1.34–2.76	<0.001
Antibiotics are like painkillers	True	1		
	False	1.5	1.02–2.24	0.041
	I do not know	1.1	0.79–1.61	<0.501
A patient is admitted	I am confident they will get well	1		
	Not confident	0.9	0.75–1.18	0.582
	It depends on the sickness	0.4	0.21–0.59	<0.001
	I do not know	1.3	0.66–2.43	0.483

## Discussion

This study assessed the public consumption of antibiotics and the associated sociodemographic factors among residents of selected communities in the Lusaka district of Zambia. Of the 2038 participants, 2021 (99.2%) had used antibiotics in the last 12 months during data collection. The rate of appropriate use of antibiotics was 40.9% and associated with being female, being aged above 35 years, attaining secondary school or tertiary education, having a monthly expenditure of 195 USD and above, being aware that antibiotics were not the same as painkillers, and being confident that when someone was hospitalized, they would get well. A total of 59.1% of the participants used antibiotics inappropriately.

Our study found a high use of antibiotics (99.2%) among the residents of the Lusaka district. The high use of antibiotics in our study is evidenced by the high use of antibiotics (79.1%) in healthcare facilities and access to antibiotics without prescription (20.9%). These findings corroborated reports from a study that was conducted among communities of Ilala, Kilosa and Kibaha districts of Tanzania, where 99% of residents were reported to have used antibiotics.^123^ These results could be partially attributed to the easy antibiotic access, as reported by earlier studies.^31,94,124–126^ Additionally, the high use of antibiotics could be attributed to increased prescribing of antibiotics in healthcare facilities.^127,128^ The high use of antibiotics in healthcare facilities continues to be reported across the world.^129–132^

The high use of antibiotics reported in our study could also be attributed to self-medication (SM) practices among the residents of the sampled communities. SM practices were also reported to be 55.2% in Vietnam, 45.7% in Bangladesh, and 36.1% in Ghana.^133^ However, lower SM practices were reported in Mozambique (8%), Thailand (3.9%) and South Africa (1.2%).^133^ Community members tend to practise SM because it is more convenient than going to healthcare facilities, cheaper, and less time-consuming than going to the hospital.^133^ The use of antibiotics reported in our study was higher than the 64.2% reported in Bosnia and Herzegovina^134^ and 38.4% in Nepal.^45^ It is evident that the high consumption of antibiotics predisposes individuals to AMR infections.^124^ Hence, there is a need to reduce these practices to avoid the emergence of AMR and its consequences.

The present study found that the proportion of appropriate use of antibiotics among the study participants was 40.9%, translating into 59.1% of inappropriate use of antibiotics in the sampled communities. The appropriate use of antibiotics in our study is slightly higher than the 33% reported at the University Teaching Hospitals in Lusaka, Zambia.^116^ The low appropriate use of antibiotics in our study could be due to increased access to antibiotics without prescriptions, non-completion of antibiotic courses by community members, and the taking of leftover antibiotics. A recent study conducted in Southwest China on antibiotic prescribing patterns at children’s outpatient departments of primary care institutions concluded that for over 37 284 visits, only 18.3% of antibiotic prescriptions were appropriate.^135^ A study in Kuwait reported that 36% of the sampled population had not finished the course of treatment, and 27.5% practised SM with antibiotics to treat mainly common cold, sore throat and cough.^136^ A survey among residents of sub-Saharan African countries reported high SM practices and access to antibiotics without a prescription.^24^ However, the level is noticeably lower compared with a previous study done in Zambia where there was 100% access to antibiotics without a prescription.^94^ Consequently, the population that access antibiotics through community pharmacies tends to be lower than the one that does so through public healthcare facilities.

A study in Ethiopia reported 62.1% appropriate use of antibiotics among community members of Yirgalem town, Sidama regional state, with 37.9% of the residents having used antibiotics inappropriately due to long delays in obtaining services at healthcare facilities, busy day programmes, and cutting costs of medication.^137^ These practices of accessing antibiotics without a prescription, non-adherence to completion of antibiotic courses, and taking leftover antibiotics have been reported in other studies.^138,139^ A study in Ghana reported 86.6% inappropriate use of antibiotics, which was due to the community members buying antibiotics using their out-of-pocket money, seeking healthcare services outside hospitals/clinics, seeking medical help in pharmacies, and buying antibiotics in instalments.^140^ A multinational study involving Cambodia, Madagascar and Senegal found that 76.5% of antibiotics for outpatients were inappropriately prescribed.^141^ There is also a notable and higher prevalence of SM with antibiotics without a prescription among community pharmacies in South Africa.^142^ Consequently, the inappropriate use of antibiotics is a driver of AMR and requires urgent community education and engagement.^51,137,143–145^

Our multivariable logistic regression found that the appropriate use of antibiotics was associated with being female, being aged above 35 years, attaining secondary school or tertiary education, having a monthly expenditure of 195 USD and above, being aware that antibiotics were not the same as painkillers like paracetamol and diclofenac, and being confident that when someone was hospitalized, they would get well. Based on our findings, females tend to use antibiotics more appropriately than males, indicating better compliance with instructions on the medication and avoidance of SM practices. These findings corroborate those reported in another study.^137^ This is because females tend to have better health-seeking behaviour compared with males.^146^ Similar to our findings, other studies have demonstrated that older age was associated with the appropriate use of antibiotics,^147^ while younger populations tend to use antibiotics inappropriately.^137,148^ This also could indicate poor health-seeking behaviour among the young population and reduced income to access medical services. Evidence has shown that individuals with a low education level tend to misuse antibiotics more compared with those with a high education level.^147,149^ However, attaining higher education must be accompanied by a change in behaviour for individuals to appropriately utilize antibiotics.^150^

Consequently, income may affect access to antibiotics as those from low-income settings tend to buy short courses of antibiotics and are unable to access medical services from hospital facilities, thereby resorting to SM and contributing to inappropriate use of antibiotics.^27,140,149^ Intriguingly, community members with adequate finances can seek medical help and purchase the required courses of antibiotics when prescribed.^27^ Our findings demonstrate the impact of sociodemographics on the appropriate use of antibiotics among community members.

We are aware that our study had limitations. First, our study was conducted in one district of Lusaka province; hence, the results may not be generalizable for the entire province and the country. Second, this study used a cross-sectional study design that is prone to recall bias. Additionally, a question concerning the use of antibiotics in the last 12 years may also lead to recall bias. Finally, we did not collect information on the actual conditions that led to the use of antibiotics. However, the study provided critical information on the consumption of antibiotics and associated predisposing factors among community members, and this can form the basis for developing and implementing interventional strategies.^74,77–79,151^ Additionally, educational activities may be used to promote the awareness and knowledge of community members of AMR. Intriguingly, the findings of this study may be used to promote community engagement in the fight against AMR.

### Conclusions

This study found high consumption of antibiotics among the community members of the Lusaka district of Zambia, with most antibiotics accessed through the hospitals and clinics. The appropriate use of antibiotics was low among the study participants. Our study found that appropriate use of antibiotics was associated with being female, an age of 35 years and above, attaining secondary school or tertiary education, having a monthly expenditure of 195 USD and above, being aware that antibiotics were not the same as painkillers, and being confident that when someone was hospitalized, they would get well. To address the low appropriate consumption of antibiotics found in this study, there is a need to promote educational campaigns on the appropriate use of antibiotics, improved antibiotic prescribing practices, and heightened regulations on access to antibiotics without a prescription. Additionally, AMS programmes should be strengthened in hospitals and clinics to ensure rational prescribing and use of antibiotics. Finally, there is a need to enhance community engagement in the fight against AMR.

## Supplementary Material

dlae034_Supplementary_Data
