# Supplementary material for: Community antibiotic consumption and associated factors in Lusaka district of Zambia: findings and implications for antimicrobial resistance and stewardship
Source: JAC Antimicrob Resist. 2024 Mar 5;6(2):dlae034. doi: 10.1093/jacamr/dlae034 (PMC10914457; doi:10.1093/jacamr/dlae034)
Supplement: dlae034_Supplementary_Data [file dlae034_supplementary_data.docx]

**Study Questionnaire**

## **Community Antibiotic Consumption and Associated Factors in Lusaka District of Zambia: Findings and Implications on Antimicrobial Resistance and Stewardship**

**Section A: Sociodemographics of participants**

1. **What is your gender?**
2. Male
3. Female
4. **How old are you (years)?**
5. 18-25
6. 26-30
7. 31-35
8. Above 35
9. **What is your marital status?**
10. Unmarried
11. Married

1. **What is your level of education?**
2. Up to primary level
3. Secondary and above
4. **What is your monthly expenditure?**
5. Below 195 USD
6. 195 USD and above
7. **Where do you live?**
8. Mtendere
9. Kaunda square
10. Chelstone
11. Kalingalinga
12. Chainda
13. Chipata
14. Ng’ombe
15. Matero
16. George
17. Kanyama
18. Chawama

**Section B: Questions on antibiotic consumption**

1. **Have you ever used antibiotics in the last 12 months?**
   1. Yes
   2. No
2. **Where did you obtain your last antibiotics?**
   1. NA (Neither suffer nor use antibiotic)
   2. Health facility
   3. Leftover antibiotics from the previous treatment
   4. Pharmacy
   5. Do not know or do not remember
3. **How did you obtain your last antibiotic used?**
4. Prescribed by a doctor or a clinical officer and dispensed by a pharmacist
5. Recommended and supplied by a pharmacist or drug retailer without a prescription
6. Self-medicated (you indicate to the pharmacist what drug you want)
7. **When did you stop taking the last antibiotics you purchased?**
8. When my illness was better
9. When I got a full course as prescribed by a doctor or clinical officer
10. I do not remember
11. **How did you obtain the last antibiotics you took?**
12. Prescribed by a doctor/ clinical officer and dispensed by a pharmacy professional
13. Not prescribed by a doctor or a clinical officer
14. **How did you take your antibiotics?**
15. Taken as prescribed and recommended by the prescriber
16. Not prescribed nor taken as recommended by the prescriber
17. **Antibiotics are the same as Dolaren, Paracetamol, and Diclofenac?**
18. True
19. False
20. I do not know
21. **When a patient is admitted to a hospital, are you confident that they will get well and be discharged?**
22. I am confident they will get well
23. Not confident
24. It depends on the sickness
25. I do not know

***Thank you very much for your participation***
